# Electromagnetic Wave Propagation in Body Area Networks Using the Finite-Difference-Time-Domain Method

**DOI:** 10.3390/s120709862

**Published:** 2012-07-23

**Authors:** Jonathan N. Bringuier, Raj Mittra

**Affiliations:** 1 Department of Electrical Engineering, The Pennsylvania State University, University Park, PA 16802, USA; 2 King Fahd University of Petroleum and Minerals, Dhahran 31932, Saudi Arabia; E-Mail: mittra@engr.psu.edu

**Keywords:** Body Area Networks, FDTD, conformal, numerical phantoms, Cole-Cole model, recursive convolution method, mutual coupling

## Abstract

A rigorous full-wave solution, via the Finite-Difference-Time-Domain (FDTD) method, is performed in an attempt to obtain realistic communication channel models for on-body wireless transmission in Body-Area-Networks (BANs), which are local data networks using the human body as a propagation medium. The problem of modeling the coupling between body mounted antennas is often not amenable to attack by hybrid techniques owing to the complex nature of the human body. For instance, the time-domain Green's function approach becomes more involved when the antennas are not conformal. Furthermore, the human body is irregular in shape and has dispersion properties that are unique. One consequence of this is that we must resort to modeling the antenna network mounted on the body in its entirety, and the number of degrees of freedom (DoFs) can be on the order of billions. Even so, this type of problem can still be modeled by employing a parallel version of the FDTD algorithm running on a cluster. Lastly, we note that the results of rigorous simulation of BANs can serve as benchmarks for comparison with the abundance of measurement data.

## 1. Introduction

As wireless technologies continue to evolve, the development of personalized devices which exchange data with unprecedented ease and efficiency is certain to grow unabated. Recently, such technologies have garnered much attention from those interested in biomedical sensing and Body-Area-Networks (BANs). These technologies, which are data networks that operate through wireless transmission using the human body as a propagation medium, continue to be an active area of research in fields such as remote health monitoring and medical biosensors. However, the complexity of integrating efficient communication systems in the human body environment poses many challenges to the future of this emerging technology. The excitation of surface and space waves from radiating antennas mounted on the body can have a large impact on the performance of co-site body-centric antenna systems. Moreover, the presence of multiple antennas on the body can lead to unexpected coupling due to creeping wave interaction. Fortunately, recent developments in powerful numerical methods, such as the Finite-Difference-Time-Domain (FDTD) method running on parallel platforms, has made it feasible for us to carry out a detailed study of these body-centric antenna systems.

## 2. Simple Models for BANs

For many years researchers have used simplified geometries to model the interaction of electromagnetic energy with biological tissue. Such geometries typically lend themselves to simple shapes, such as cylinders, ellipses and spheres for which past research has typically treated these as perfectly conducting objects, low loss high dielectrics, and surface impedance models. This type of treatment was convenient due to the availability of well-known analytical methods used solve these problems, such as the uniform theory of diffraction (UTD), ray tracing (RT), creeping waves, and eigenfunction analysis [1,2]. Indeed, these models have been shown in recent years to yield reasonably good results for simple cases that compare well to those derived numerically rigorously with modern CEM techniques. However, the asymptotic techniques fail to perform well when the dielectric medium they deal with is arbitrarily shaped, inhomogeneous and lossy, and such problems must be handled by using general-purpose numerical methods, such as the FDTD [3–6]. Nevertheless, these simple geometries can still provide a computationally efficient way to study the propagation around and through the human body. Therefore, these models remain useful for studying BANs.

In this section we provide the results of a simplified model for the human body torso. The proposed model is a 3-layer elliptical structure having major and minor axis of 150 cm and 120 cm, respectively. This model has been used in [7] to investigate the coupling around a 2-D ellipse using the sub-band FDTD, UTD/RT and measurement techniques at UWB frequencies. However, in that work the model was assumed to be perfectly conducting when applying the UTD/RT method while a homogeneous muscle phantom was employed for the sub-band FDTD analysis. Additionally, a conformal FDTD algorithm was not used to improve the accuracy of the curved surface whereas the author has done so in this work. Furthermore, it neither accounted for the multi-layer dielectric properties of the tissue in the human torso, nor did it simulate the actual radiating element, *i.e.*, viz., the monopole antenna rigorously.

The 3-layer ellipse model incorporates the skin, fat, and muscle layers. In [8] a simple 3-layer planar slab model using 3 mm skin layer, 5 mm fat layer and muscle was used to study the penetration depth of an incident plane wave for use in implantable medical devices. In this work we have used the same thickness for the skin and fat layers in the elliptical model.

Although the FDTD method enjoys a significant advantage over MoM in terms of its ability to simulate complex structures and lossy inhomogeneous materials, it has been known to require far more computational resources than are usually available to accurately model, simultaneously, the fine features of the radiating element, the layered structure of the geometry and the electrically large size of the entire structure typically encountered in the study of BANs. To circumvent this problem, most research done on BANs by utilizing the FDTD have been carried out using a point source approximation. While such an approximation may be adequate for directly estimating the path loss associated with the body, it does not rigorously account for a number of crucial antenna factors that affect the antenna performance, such as finite ground size, radiation pattern and efficiency. Furthermore, any field behavior in either the near or the intermediate region is inherently neglected in the point source approximation, whereas the physical structure of the radiating element must be included to properly model the physical system.

In this paper we have used a parallel version of FDTD that can handle such electrically large geometries as well as the fine features of the radiating element. To this end, we have used quarter-wave monopoles resonant at 2.45 GHz with a 75 mm × 75 mm square ground plane located at tangent points one to two FDTD cells away from the models analyzed in this study. Although other antennas could be used, the choice of the monopole antenna has the distinct advantage in that the radiation pattern in the plane azimuthal to the monopole is inherently stable across the bandwidth of interest. However, the monopoles used in this study do not have an impedance matching bandwidth wide enough to cover the frequencies of interest, and, therefore, the channel path loss data between observation points should be considered relative to one another for a given frequency. Nevertheless, the polarization and radiation pattern deviations of the transmitter and receivers across the frequency band can more or less be neglected when performing coupling calculations, which make these antennas useful for studying channel characteristics of the on-body propagation medium.

**Figure 1.**
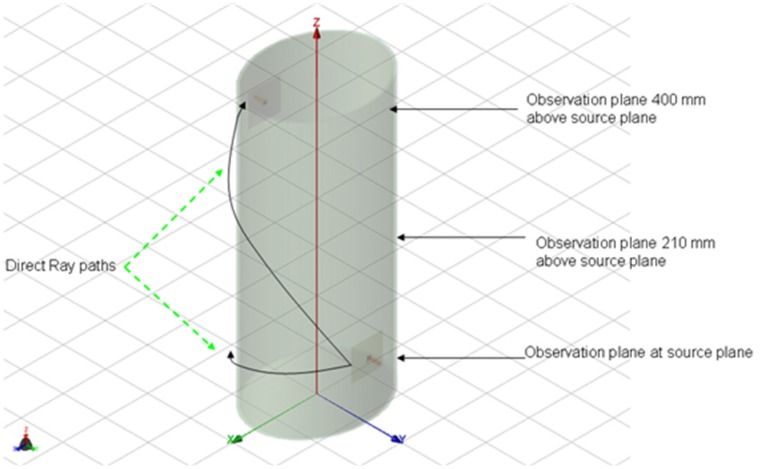
3-layer ellipse model of the human torso with transmitting antenna at the front and receiving antenna at the back.

**Figure 2.**
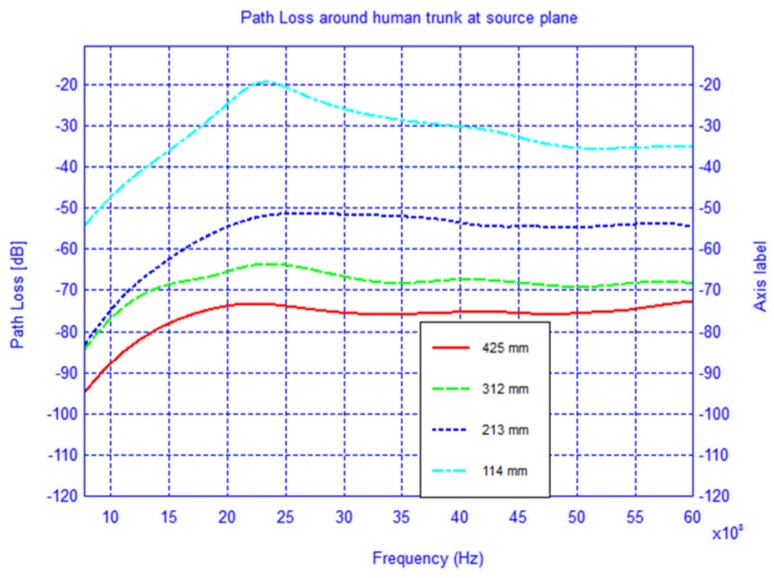
Path loss around the cylindrical human trunk model at the source plane.

**Figure 3.**
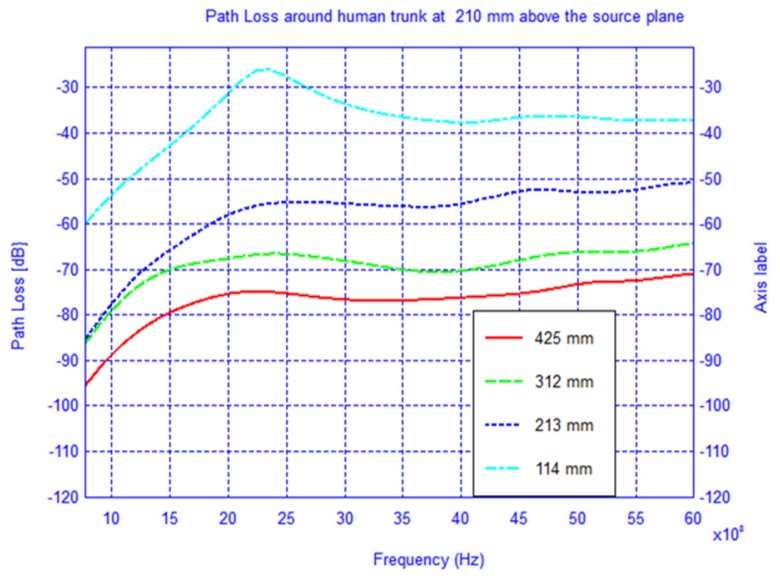
Path loss around the cylindrical human trunk model 210 mm above source plane.

**Figure 4.**
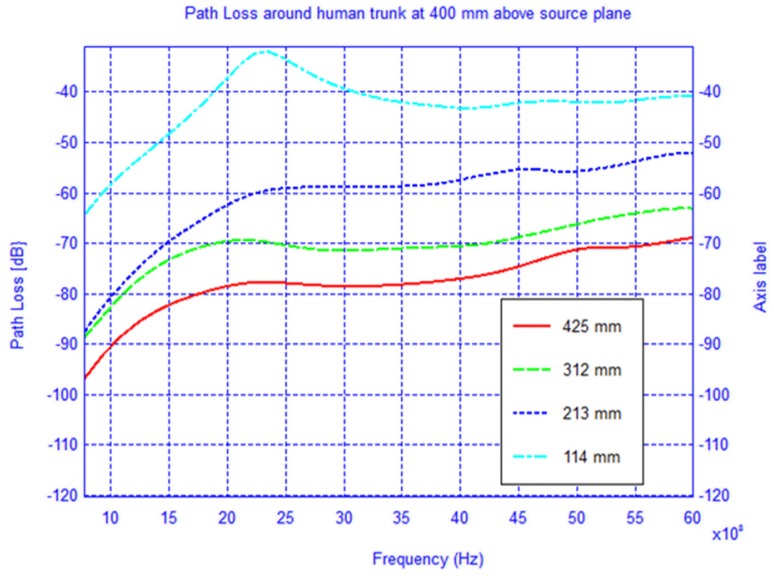
Path loss around the cylindrical human trunk model 400 mm above source plane.

The setup used in the simulation is shown in Figure 1 where the receiver is located first in the source plane and then displaced vertically by 210 mm and 400 mm, respectively, from the above plane. For each observation plane the receiver is moved around the elliptical trunk model and the *S*_21_ is recorded. The mode of propagation is known to be a creeping wave and the direct ray paths are shown. For each observation plane the *S*_21_ was calculated along the elliptical path in the level plane and plotted for frequencies between 0.8–6 GHz as shown in Figures 2–4.

**Figure 5.**
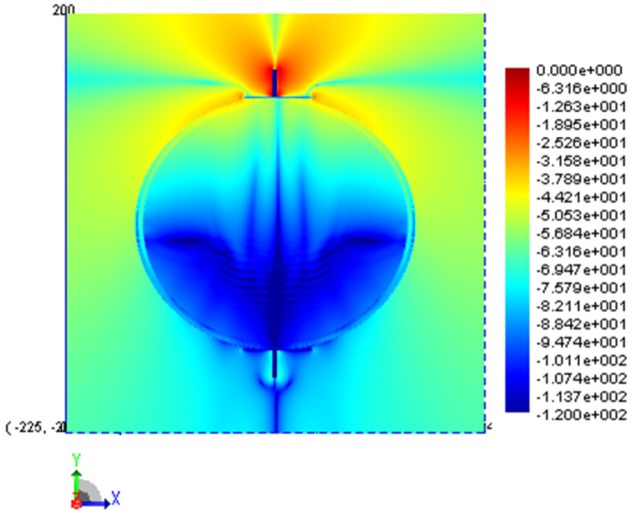
Electric field distribution in the source plane.

**Figure 6.**
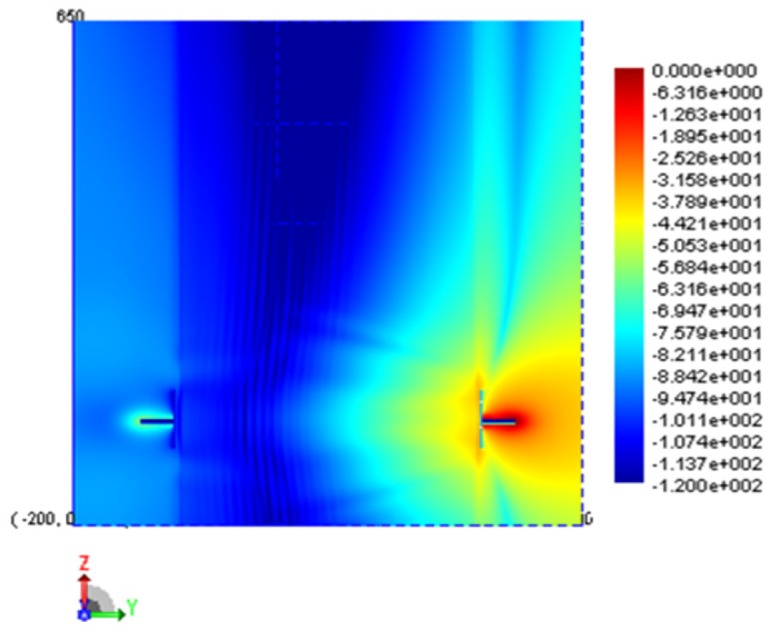
Electric field distribution on a vertical cut plane bisecting the cylindrical model.

For BANs it is typically assumed that the wave propagation through the body is so attenuated that its contribution can be ignored. To verify this we have plotted the fields for the cross-section of the ellipse in the source plane as well as a vertical cross-section along the extent of the ellipse model. The electric field plots are shown in Figures 5 and 6. From these figures it can be seen that the field magnitude inside the model is less than −100 dB and the main source of energy transfer is along the surface of the trunk in the form of a creeping wave.

**Figure 7.**
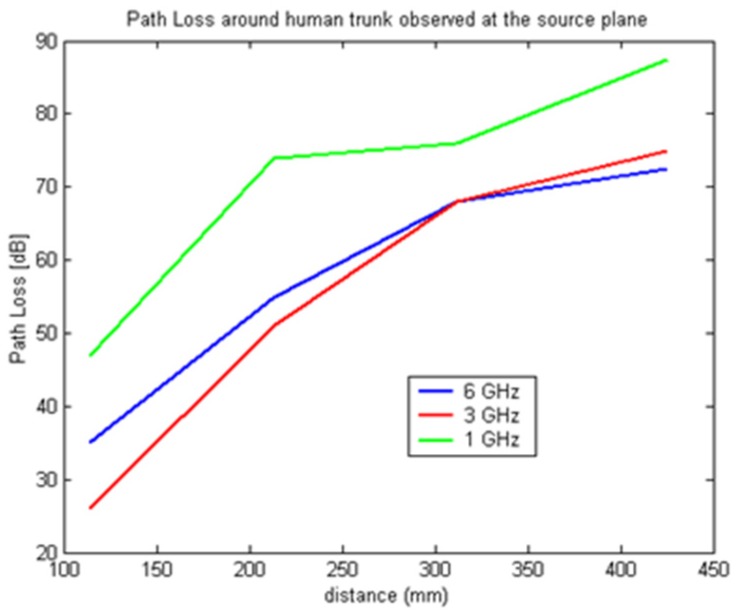
Path loss *versus* separation distance with receiving antenna at the source plane.

**Figure 8.**
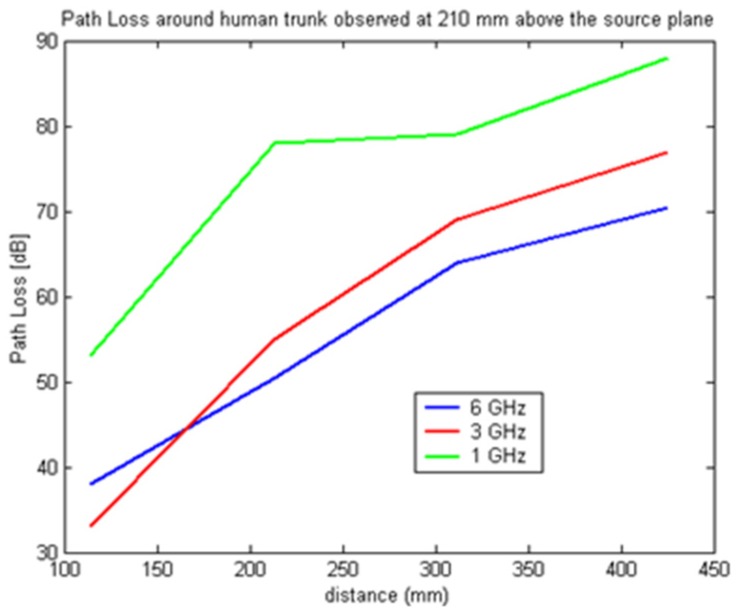
Path loss *versus* separation distance with receiving antenna 210 mm above the source plane.

**Figure 9.**
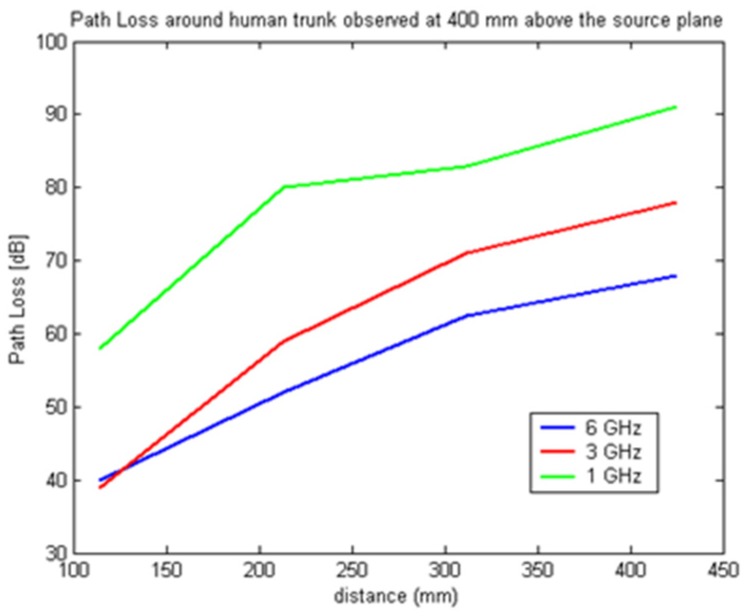
Path loss *versus* separation distance with receiving antenna 400 mm above the source plane.

In Figures 7–9 the path loss is plotted *versus* the distance around the elliptical model in each of the different observation planes. As shown in Figure 7, which depicts the observations made in the source plane, the attenuation at higher frequencies exhibit a clear monotonic behavior and is characteristic of a creeping wave. The results in Figures 7–9 have been found to agree closely with those in the literature for other body models [1,2,7,9–19]. These results can be useful as reference points when approximating the rate of attenuation along the trunk in body-centric network scenarios.

## 3. Numerical Phantoms for BANs

**Figure 10.**
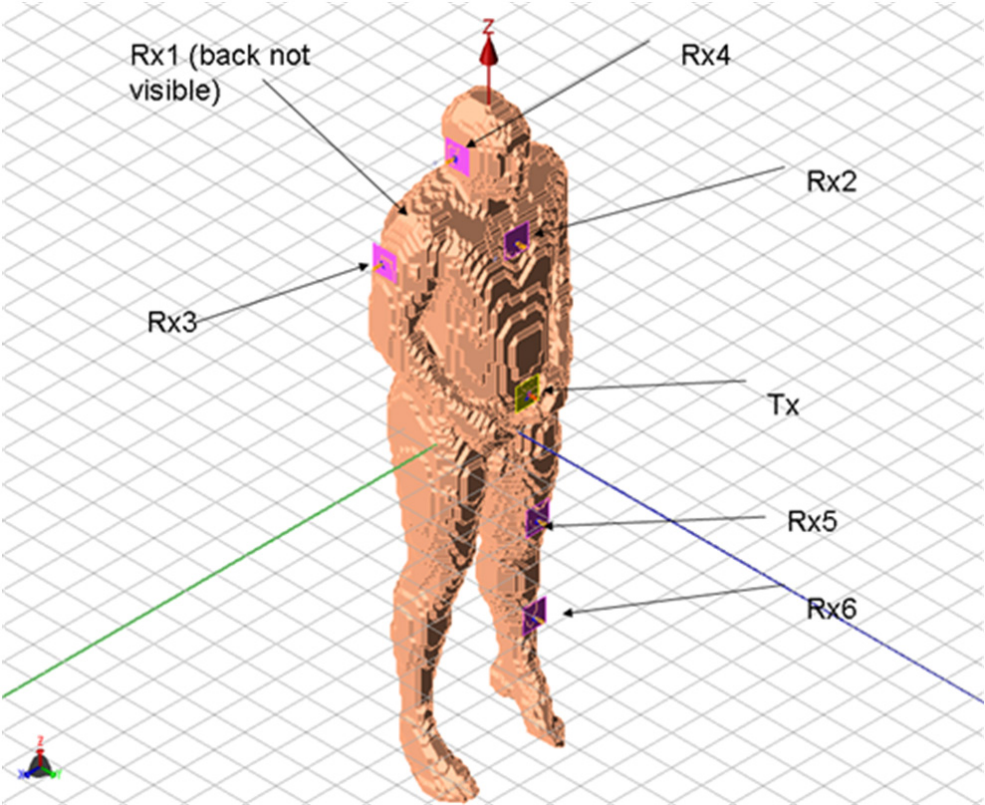
Numerical human body phantom based on 3D CT scan voxel set with transmitting and receiving antennas in typical BAN scenario.

Although simplified geometries have their use in analyzing body-centric communication networks, the availability of realistic human body models enables us to use a more rigorous approach to understand how the irregular shape of the body affects the performance of BANs. One of the most commonly used body models is shown in Figure 10, where the original data set is comprised of voxels (*i.e.*, 3D matrix data set). Furthermore, a hypothetical BAN configuration is shown with the transmitting and receiving antennas. To reduce the computational resources needed in the simulation, a lossy homogeneous model was used.

**Figure 11.**
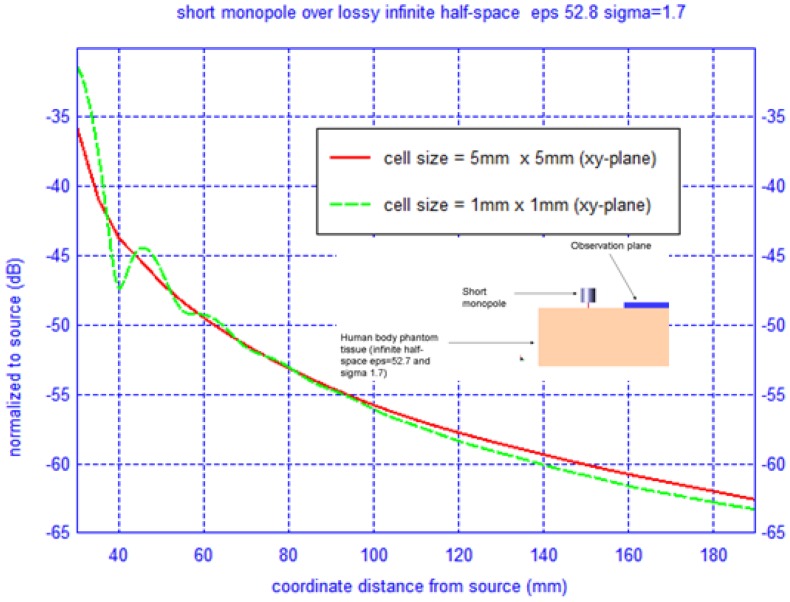
Experiment to determine if the down-sampled human body voxel set causes numerical inaccuracies (*λ* = 10 *cm*).

Additionally, the resolution of the original voxel data set, which is 1 mm × 1 mm × 1 mm, was down-sampled to 3 mm × 3 mm × 3 mm. Even at this lower resolution the computational resources needed are high, specifically 8 hours on 16 Pentium 4 CPUs. Although this may seem too coarse for the conventional λ/20 cell size, the fields couple on or near the surface of the body, where the mesh can be coarser, and we are interested in separation distance well into the asymptotic region of the transmitting antenna. To demonstrate that the loss of accuracy is negligible in doing so, we have performed a simple experiment by computing the difference in field magnitude at 3 GHz—far away from the transmitting antenna on the same plane as shown in Figure 11. It is evident from the results that in the asymptotic region there is little loss in accuracy by utilizing a down-sampled phantom model.

## 4. Dielectric Properties and Dispersion Models of Biological Tissue

It is commonly claimed in the literature that a good approximation for the body can be made by using a full muscle phantom or a two-thirds muscle equivalent phantom, the latter being more commonly used in EMC analysis for SAR [20,21]. However, realistic biological tissues exhibit dispersion characteristics that are not of the traditional Debye type used in the FDTD model, whose spectral behavior is usually represented by the form:

(1) $$\hat{ɛ}\left(\omega \right)={ɛ}_{\infty }+\sum_{n=1}^{N}\frac{\Delta {ɛ}_{n}}{1+j\omega \tau_{n}}+\frac{\sigma_{n}}{j\omega {ɛ}_{0}}$$

where *τ_n_* is the relaxation time constant Δ*ε_n_* is the spectral coefficient and *σ_n_* is the static ionic conductivity. This model can be handled in FDTD through a simple convolution extension of the basic FDTD algorithm. In general, biological tissue exhibit a broadened spectrum which is empirically accounted for by the parameter 0 ≤ *α_n_ <* 1, (see Gabriel *et al.* [22]) and is typically referred to as the Cole-Cole model. It is given by:

(2) $$\hat{ɛ}\left(\omega \right)={ɛ}_{\infty }+\sum_{n=1}^{N}\frac{\Delta {ɛ}_{n}}{1+{\left(j\omega \tau_{n}\right)}^{1-\alpha_{n}}}+\frac{\sigma_{n}}{j\omega {ɛ}_{0}}$$

The difficulty in this model lies in the fact that there are not any simple closed-form expressions amenable to the FDTD algorithm. Specifically, this is due to the fact that the inverse Fourier transform is non-trivial.

### 4.1. Dielectic Spectrum Approximation

In this Section we will introduce a technique that can be used to implement the Cole-Cole model into the desired frequency band of interest for simple geometries relevant to BANs (e.g., planar human tissue phantoms) in FDTD. Since the static conductivity term can be handled easily in the FDTD update equations we turn our attention to the term containing *α_n_*. First we postulate that each term in the Cole-Cole model can be rewritten as an integral representation of a Debye spectrum as follows:

(3) $$\frac{{\hat{ɛ}}_{i}\left(\omega \right)}{\Delta {ɛ}_{i}}=\frac{1}{1+{\left(j\omega \right)}^{1-\alpha_{i}}}=\frac{1}{1+{\left(j\omega \right)}^{\beta_{i}}}=\int_{0}^{\infty }\frac{g_{i}\left(\lambda \right)}{\lambda +j\omega }d\lambda$$

Then the expression for the electric field density follows as:

(4) $$D\left(\omega \right)={\hat{ɛ}}_{i}\left(\omega \right)E\left(\omega \right)$$

(5) $$=\Delta {ɛ}_{i}\int_{0}^{\infty }\frac{E\left(\omega \right)g_{i}\left(\lambda \right)}{\lambda +j\omega }d\lambda$$

where by the inverse Fourier transform yields:

(6) $$D\left(t\right)=F^{-1}\left\{{\hat{ɛ}}_{i}\left(\omega \right)E\left(\omega \right)\right\}\left(t\right)=\int_{0}^{\infty }E\left(t\right)⊗e^{-\lambda t}g_{i}\left(\lambda \right)d\lambda$$

(7) $$D\left(t\right)=E\left(t\right)⊗L\left\{g_{i}\left(\lambda \right)\right\}\left(t\right)$$

Therefore, if it is possible to find a time domain representation for the Laplace transform of the unknown spectrum *g_i_*(*λ*) and, hence, we may extend the basic convolutional FDTD to implement non-Debye types of materials.

The problem at hand is to determine a suitable function for which we can derive the Laplace transform. To do this we first rewrite the Debye integral representation as:

(8) $$\frac{\hat{ɛ}\left(\omega \right)}{\Delta ɛ}=\int_{0}^{\infty }\frac{g\left(\lambda \right)}{\lambda +j\omega }d\lambda =\int_{0}^{\infty }g\left(\lambda \right)\int_{-\infty }^{\infty }e^{-j\omega \rho }e^{-\lambda \rho }u\left(\rho \right)d\rho d\lambda$$

(9) $$=\int_{0}^{\infty }\int_{0}^{\infty }g\left(\lambda \right)e^{-\lambda \rho }e^{-j\omega \rho }d\lambda d\rho$$

where *u*(*ρ*) is the Heaviside step function. Now by Fubini's Theorem:

(10) $$\frac{\hat{ɛ}\left(\omega \right)}{\Delta ɛ}=\int_{0}^{\infty }e^{-j\omega \rho }\left[\int_{0}^{\infty }g\left(\lambda \right)e^{-\lambda \rho }d\lambda \right]d\rho$$

To find *g*(*λ*) we expand it in terms of some complete basis set for which we know the Laplace transform. Specifically, we chose Bessel functions of the first kind which form a complete set in ℜ: *λ* ∈ [0, ∞). Therefore, we have:

(11) $$g\left(\lambda \right)=\sum_{n=0}^{\infty }C_{n}J_{n}\left(a\lambda \right)$$

where *a* is a fitting parameter. Obviously we must truncate this infinite series for numerical purposes; hence we write:

(12) $$g\left(\lambda \right)\approx \sum_{n=0}^{N}C_{n}J_{n}\left(a\lambda \right)$$

Now we use the Laplace transform relation given by:

(13) $$\int_{0}^{\infty }J_{n}\left(a\lambda \right)e^{-\lambda \rho }d\lambda =\frac{1}{\sqrt{\rho^{2}+a^{2}}}\frac{a^{n}}{{\left(\rho +\sqrt{\rho^{2}+a^{2}}\right)}^{n}}$$

Of course, we could have used Equation (1) directly in Equation (5); however, we have found that the integral converges much more slowly than that of the reformulation given in Equation (10). After inserting Equation (13) into the equation given in Equation (10) we have:

(14) $$\frac{\hat{ɛ}\left(\omega \right)}{\Delta ɛ}=\int_{0}^{\infty }e^{-j\omega \rho }\left(\sum_{n=0}^{N}C_{n}\frac{1}{\sqrt{\rho^{2}+a^{2}}}\frac{a^{n}}{{\left(\rho^{2}+\sqrt{\rho^{2}+a^{2}}\right)}^{n}}\right)d\rho$$

Next, we can apply the method of least squares to find the coefficients *C_n_*. However, from Equation (7) it is obvious that we must require the *C_n_*'s to be real. Therefore we have a constrained least squares system of equations given by the following:

(15) b¯T=[Re∫0∞ɛ^(ω)ΔɛI0dωIm∫0∞ɛ^(ω)ΔɛI0dωRe∫0∞ɛ^(ω)ΔɛI1dωIm∫0∞ɛ^(ω)ΔɛI1dω••Im∫0∞ɛ^(ω)ΔɛINdω]

(16) $${\bar{x}}^{T}=\left[\begin{array}{ccccccc} C_{1} & C_{2} & C_{3} & • & • & • & C_{N} \end{array}\right]$$

(17) A¯¯=[Re∫0∞I0I0dωRe∫0∞I0I1dωRe∫0∞I0I2dω•••Re∫0∞I0INdωIm∫0∞I0I0dωIm∫0∞I0I1dωIm∫0∞I0I2dω•••Im∫0∞I0INdωRe∫0∞I1I0dωRe∫0∞I1I1dω•Im∫0∞I1I0dωIm∫0∞I1I1dω•••Re∫0∞INI0dω•Im∫0∞INI0dωIm∫0∞ININdω]

where *A̿* is 2NxN, *b̄* is 2Nx1, *x̄* is Nx1, $\bar{x}=\bar{\bar{A^{+}}}\bar{b}$ and

(18) $$I_{n}=\int_{0}^{\infty }\frac{e^{-j\omega \tau }a^{n}}{\sqrt{\tau^{2}+a^{2}}\cdot {\left(\tau^{2}+\sqrt{\tau^{2}+a^{2}}\right)}^{n}}d\tau$$

In common with most over-constrained inverse problems, we must use the Moore-Penrose pseudoinverse for $\bar{\bar{A^{+}}}$ to solve the system of equations. In practice, the integral cannot be evaluated over all possible frequencies; hence, it is typically replaced by a discrete summation over all frequencies of interest where the spectral match is desired.

As an illustrative example of this approach, the 4-term Cole-Cole model was taken for the tissue corresponding to the muscle. Although the preceding formulation corresponds to a single Cole-Cole term, the postulated Debye spectrum can equally well be applied to the entire summation of Cole-Cole terms. The parameters given by Gabriel [22] listed in Table 1.

**Figure 12.**
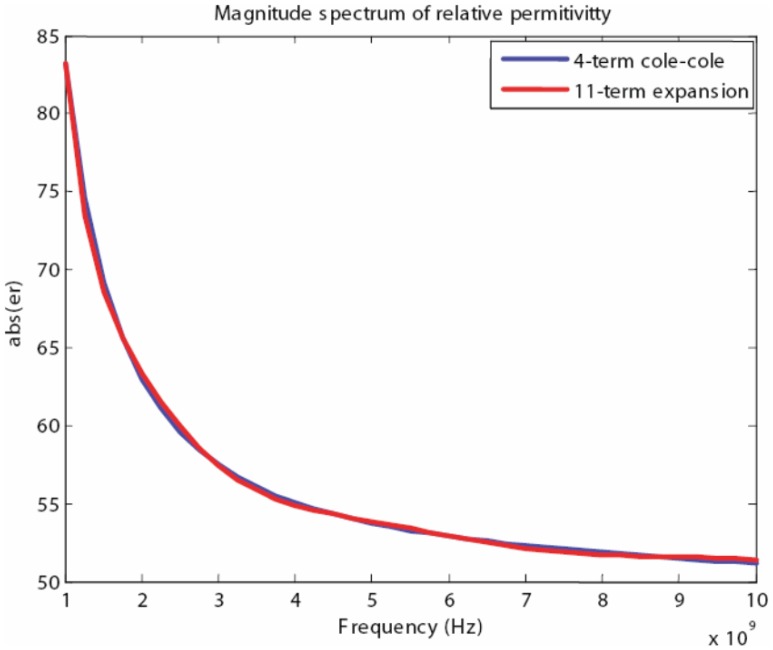
Magnitude spectrum of the relative permittivity for the 4 term Cole-Cole model and the spectrum approximation.

**Figure 13.**
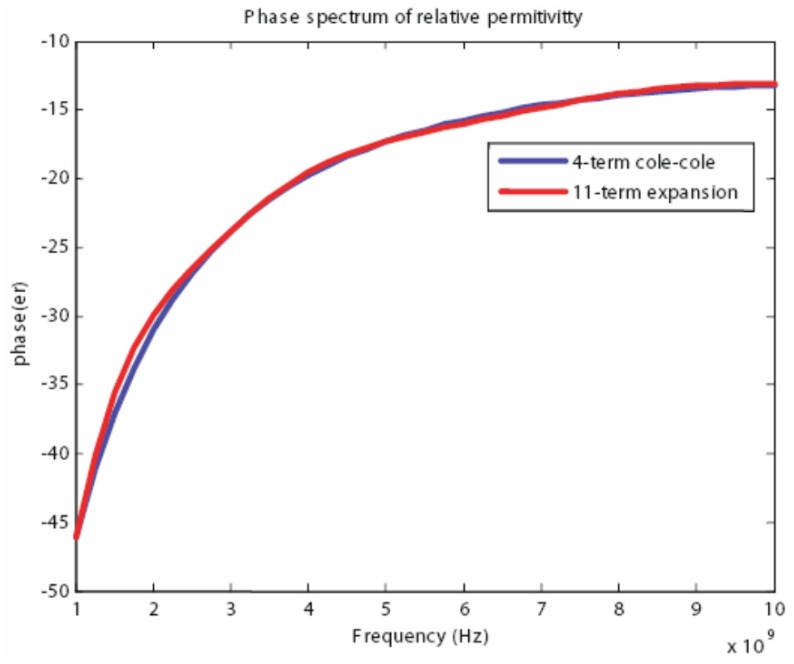
Phase spectrum of the relative permittivity for the 4 term Cole-Cole model and the spectrum approximation.

**Figure 14.**
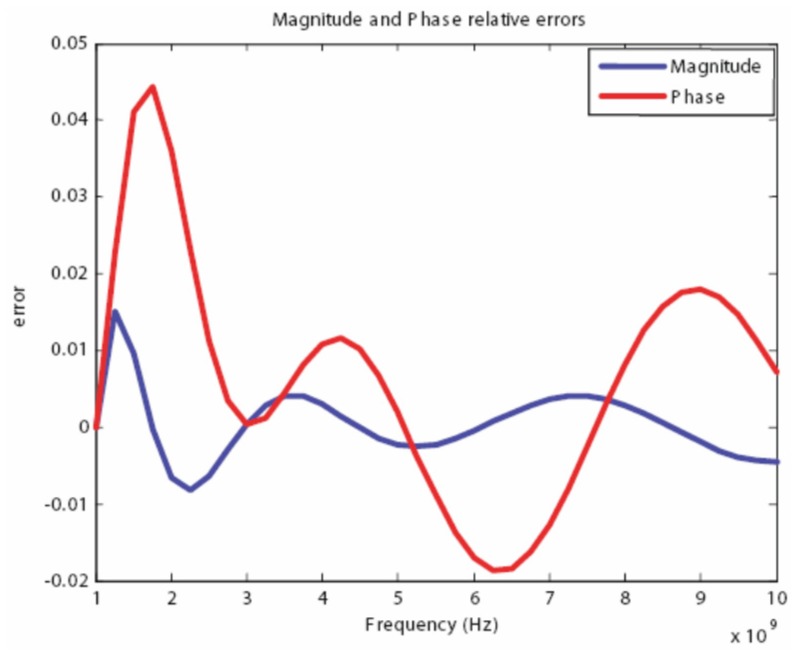
Magnitude and Phase relative errors of the spectrum approximation and the 4 Cole-Cole model.

Using the proposed algorithm we have obtained the following coefficients for an 11th-order expansion that are listed in Table 2. In addition, we have found that a good match was achieved when the fitting parameter was chosen to be *a* = 0.5*e* − 9. Although initially the order of the expansion may seem to be large, the expansion is only 2.75 times that of the original 4-term Cole-Cole model. Figures 12–13 show the magnitude and phase spectrum match for the band of interest, specifically 1–10 GHz. Furthermore, Figure 14 shows that the relative error is less than 5 percent over the entire band, for both magnitude and phase, and this can be considered a very good match for simulation purposes.

**Figure 15.**
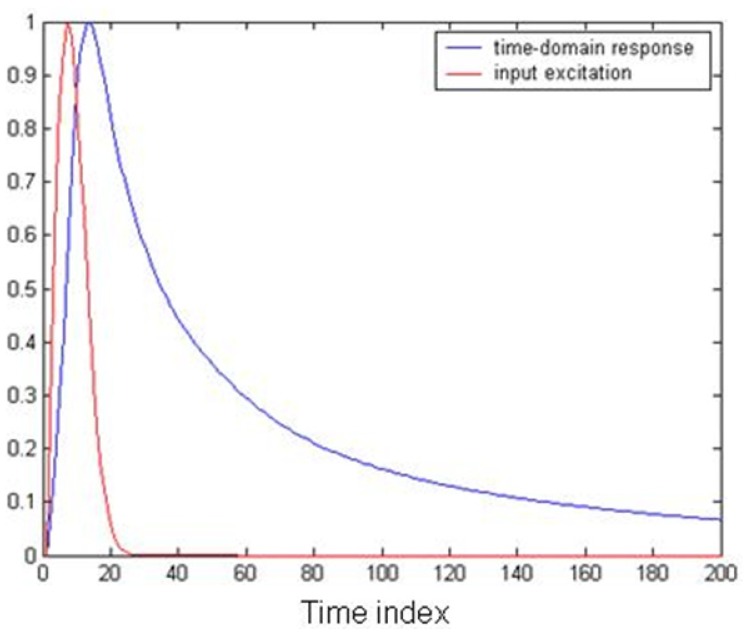
Normalized time-domain response of the electric flux density using the spectral approximation.

As an example, the calculated coefficients along with the fitting parameter were used to model the frequency response in a muscle medium. Using the convolution sum of Equation (7) for a Gaussian pulse excitation in the medium yields the result shown in Figure 15. It can be seen from Figure 15 that the electric flux density inside the muscle medium is influenced by its frequency domain characteristics as modeled by the proposed algorithm.

### 4.2. Recursive Convolution Method for Debye Materials

The problem with incorporating the spectral approximation algorithm proposed in the previous section is that FDTD requires a record-keeping of all past time values of the electric field components. If either the problem size is small, or reflection properties from an infinite planar slab are of interest then this approach may be feasible. However, for practical problems, the computational domain can become exceedingly large (e.g., upward of 1 billion unknowns for realistic human body phantoms), and other approximation methods must be used. Often times, the dispersion properties of biological tissue can be approximated by a Debye model over a finite frequency bandwidth. In this case it is possible to formulate an FDTD updating scheme for dispersive materials that does not require keeping all past histories of the fields.

The recursive convolution FDTD is one such method and is discussed in [5]. The essential features of this method are based on the Debye dispersion model given by:

(19) $$ɛ\left(\omega \right)={ɛ}_{o}{ɛ}_{\infty }+{ɛ}_{o}\chi \left(\omega \right)$$

where *χ*(*ω*) is explicitly given as:

(20) $$\chi \left(\omega \right)=\sum_{p=1}^{P}\frac{A_{p}}{\omega -W_{p}}$$

After some extensive manipulations the final form of the FDTD updating equations for the electric field become:

(21) $${\vec{E}}^{n+1}=\frac{\frac{{ɛ}_{\text{eff}}}{\Delta t}-\frac{\sigma_{\text{eff}}}{2}}{\frac{{ɛ}_{\text{eff}}}{\Delta t}+\frac{\sigma_{\text{eff}}}{2}}{\vec{E}}^{n}+\frac{1}{\frac{{ɛ}_{\text{eff}}}{\Delta t}+\frac{\sigma_{\text{eff}}}{2}}\vec{\nabla }\times {\vec{H}}^{n+\overset{1}{\;}/\underset{2}{\;}}+\frac{\frac{{ɛ}_{o}}{\Delta t}}{\frac{{ɛ}_{\text{eff}}}{\Delta t}+\frac{\sigma_{\text{eff}}}{2}}\sum_{p=1}^{P}\psi_{p}^{n}$$

and $\psi_{p}^{n}$ is given by:

(22) $$\psi_{p}^{n}={\vec{E}}^{n}\Delta \chi_{p}^{0}+e^{jW_{p}\Delta t}\psi_{p}^{n-1}$$

The formulation given in Equation (21) can be used to incorporate materials that exhibit Debye-type dispersive properties into the FDTD. If the geometry is simple (e.g., a dispersive sphere), this method has been shown to give improved accuracy. However, in the case of the human body, where the object has irregular features, and many dispersive dielectric layers, the conformal (CFDTD) version, combined with the recursive convolution method, can lead to unstable results. In fact, when using the parameters in Table 1 with *α_i_* = 0 to simulate the body shown in Figure 10, the results were found to diverge. This problem further demonstrates the difficulties when trying to rigorously simulate a realistic human body. Therefore, we have used a common approximation [21] for the human body as consisting of a non-dispersive muscle (*ε_r_* = 52.7, σ = 1.7) or its two-thirds equivalent phantom in the band of 0.8–3 GHz.

## 5. Simulations and Results

Using the muscle and the two-thirds muscle equivalent human body phantom, the CFDTD algorithm was used to simulate the setup previously shown in Figure 10.

**Figure 16.**
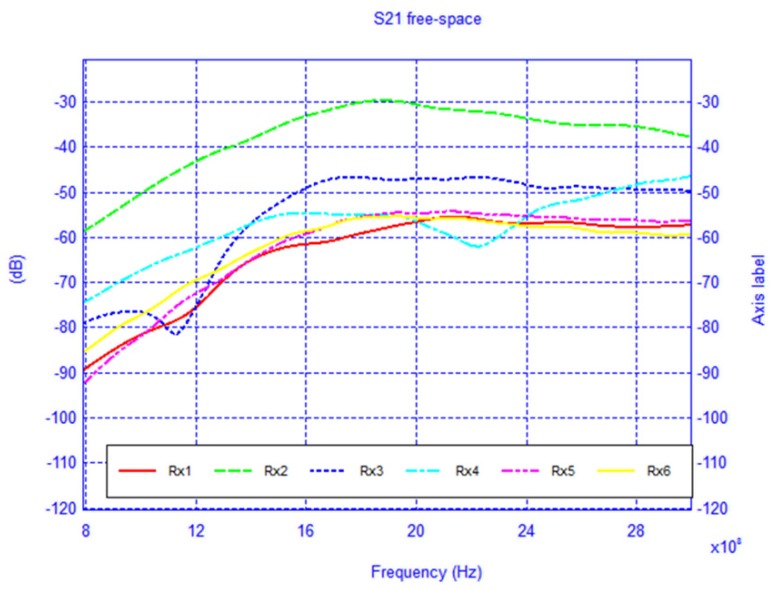
*S*_21_ of the BAN network scenario with body absent.

**Figure 17.**
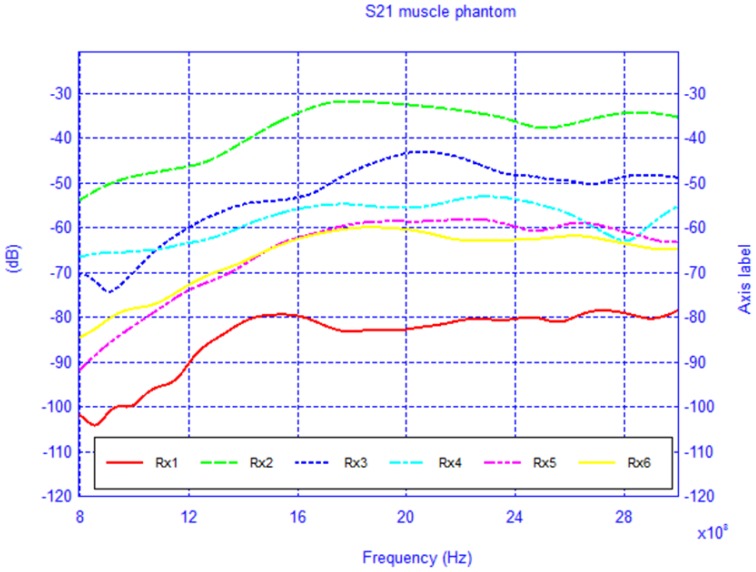
*S*_21_ of the BAN network scenario with muscle phantom.

**Figure 18.**
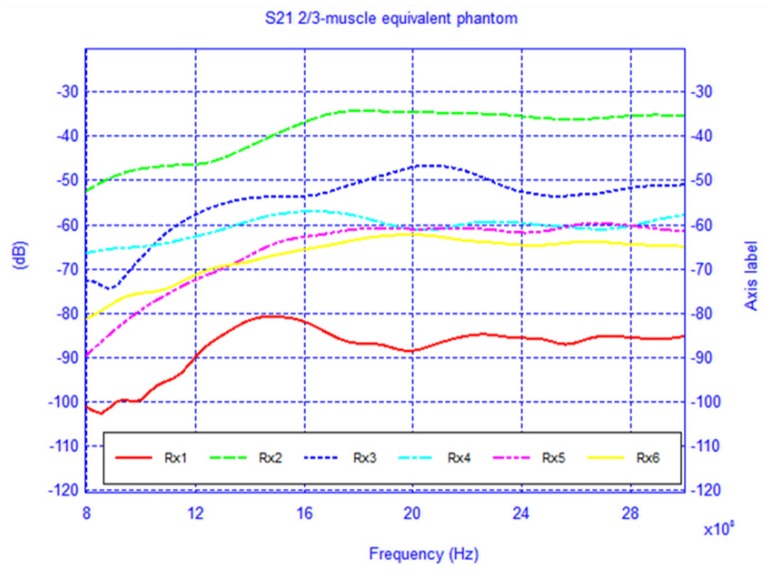
*S*_21_ of the BAN network scenario with 2/3-muscle phantom.

**Figure 19.**
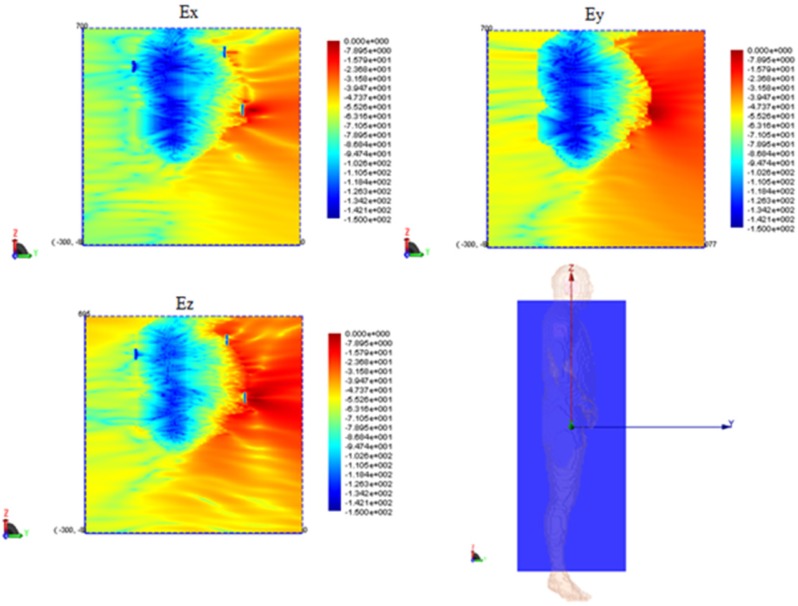
Electric field distributions (dB) on a vertical cut plane bisecting the body.

For each model, the network configuration was simulated and the *S*_21_ was calculated. Additionally, the network was also simulated in the absence of the body in order to understand the influence of its presence. The free-space case (body absent) is shown in Figure 16 and the results of the two body models are shown in Figures 17 and 18. To gain insight into the field distribution around the body the electric field is shown in Figure 19 at 3 GHz. It is evident from the results that the line-of-sight (LOS) or near LOS paths are not greatly influenced by the presence of the body for monopole antennas.

However, for the non-line-of-sight (NLOS) there is a noticeable difference in the path loss, especially at frequencies above 1 GHz, where the difference is approximately 35 dB compared to the free-space case.

**Figure 20.**
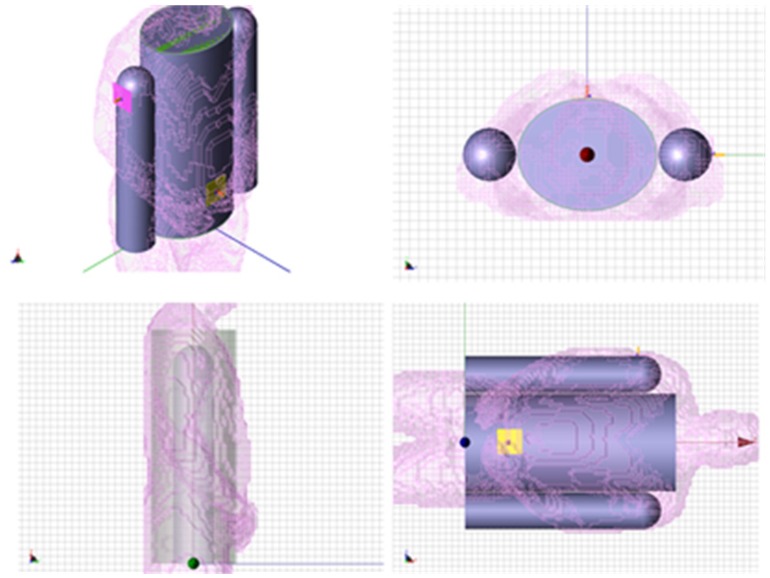
Simplified model superimposed on the human body numerical phantom.

**Figure 21.**
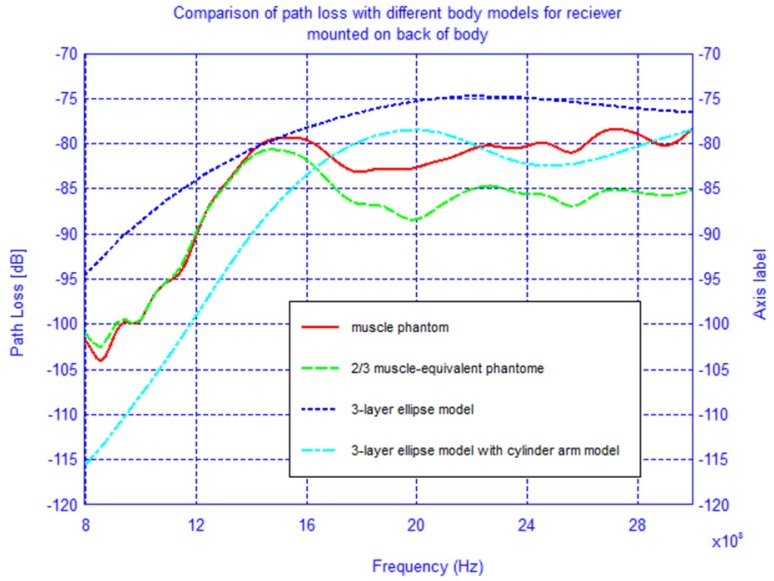
Comparison of the path loss for different phantom models with receiving antenna on the back of the body and transmitting antenna at the waist.

**Figure 22.**
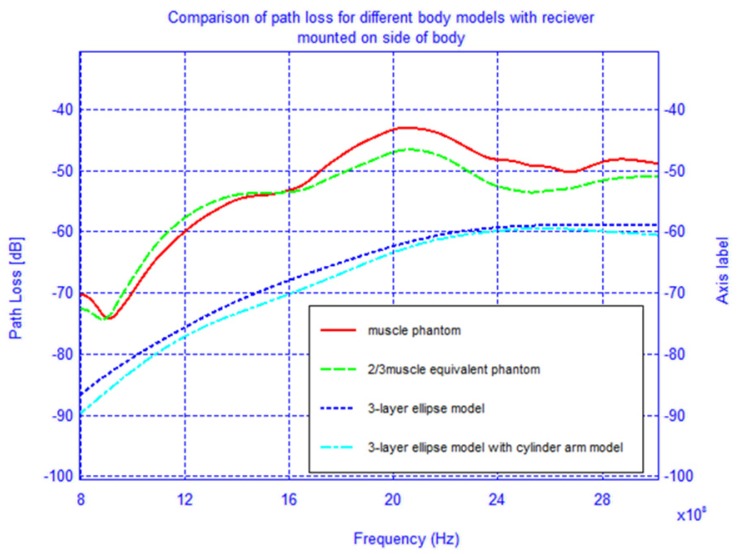
Comparison of the path loss for different phantom models with receiving antenna on the shoulder side of the body and transmitting antenna at the waist.

To determine the accuracy of the elliptical model, we compare two cases, namely when the receiver is located on the back of the body and on the shoulder-side. Additionally, in order to improve the elliptical model from the previous section, two muscle tissue arms, composed of cylinders and spheres, were placed alongside the elliptical torso model and are shown superimposed on the actual physical model in Figure 20. The results for the case when the receiver is located on the back of the body and on the shoulder-side are shown in Figures 21 and 22. For the case when the receiver is located on the back of the body the addition of the arms improves the accuracy of the results when compared to the physical model. This indicates that the arms can have a significant effect on the coupling between antennas mounted on the body. However, for the case when the antenna is placed on the shoulder side of the body, neither the elliptical model nor the ellipse with arms yielded accurate results, and there was approximately a 10 dB discrepancy between the results obtained with these and the physical models. Therefore, we surmise that the addition of other scattering features such as the head, irregular arm position and shape—not exhibited in the simplified models—play a crucial role in accurately capturing the behaviors of the fields around the human body.

## 6. Conclusions

Despite the capability of FDTD to model the complex human body in its original form, it is often desirable to seek out good geometrical approximations to simply the computational cost. This has been documented in the literature for both measurement and simulation purposes. However, to-date a quantitative study that demonstrates the accuracy of the results using simplified models has not yet been reported. A detailed characterization of this comparison has been carried out and presented in this paper. It has been shown that the rigorous FDTD phantom model yielded results that are consistent with the measurements found in the literature, and they provide valuable information into potential cosite BAN antenna systems. In addition, we have shown simple models can give reasonably accurate results for some antenna configurations, but they can fail to reproduce the mutual coupling results when the antennas are not in line-of-sight. Therefore, it is highly desirable to carry out an in-depth study of these approximations and to see how well the results based on these approximations compare to the rigorous simulation of the human body.

## Figures and Tables

**Table 1.** 4 term Cole-Cole model parameters for muscle.

Δ*ε*_1_ = 50	*τ*_1_ = 7.23*e* − 12	*α*_1_ = 0.1
Δ*ε*_2_ = 7000	*τ*_2_ = 353.68 *e* − 9	*α*_2_ = 0.1
Δ*ε*_3_ = 1.2*e*6	*τ*_3_ = 318.31 *e* − 6	*α*_3_ = 0.1
Δ*ε*_4_ = 2.5*e*7	*τ*_4_ = 2.27 *e* − 3	*α*_4_ = 0

**Table 2.** Calculated coefficients from the spectral approximation method.

**Coefficients**	**Numerical Value**
C1	0.0002e6
C2	−0.0032e6
C3	0.0394e6
C4	−0.2759e6
C5	1.1027e6
C6	−2.4578e6
C7	2.7137e6
C8	−0.5489e6
C9	−1.7652e6
C10	1.6288e6
C11	−0.4334e6
